# Narrative review of relationship between chronic cough and laryngopharyngeal reflux

**DOI:** 10.3389/fmed.2024.1348985

**Published:** 2024-04-19

**Authors:** Viktória Hránková, Tomáš Balner, Patrícia Gubová, Lucia Staníková, Karol Zeleník, Pavel Komínek

**Affiliations:** ^1^Department of Otorhinolaryngology and Head and Neck Surgery, University Hospital of Ostrava, Ostrava, Czechia; ^2^Department of Craniofacial Surgery, Faculty of Medicine, University of Ostrava, Ostrava, Czechia; ^3^Department of Allergology and Clinical Immunology, University Hospital of Ostrava, Ostrava, Czechia; ^4^Department of Biomedical Sciences, Faculty of Medicine, University of Ostrava, Ostrava, Czechia

**Keywords:** chronic cough, non-productive cough, extraesophageal reflux, laryngopharyngeal reflux, cough receptors

## Abstract

Gastroesophageal reflux disease (GERD) as a possible cause of chronic cough is known for decades. However, more than 75% of patients with extraoesophageal symptoms do not suffer from typical symptoms of GERD like pyrosis and regurgitations and have negative upper gastrointestinal endoscopy. For such a condition term laryngopharyngeal reflux (LPR) was introduced and is used for more than two decades. Since the comprehensive information on relationship between chronic cough and LPR is missing the aim of this paper is to summarize current knowledge based on review of published information during last 13 years. Laryngopharyngeal reflux is found in 20% of patients with chronic cough. The main and recognized diagnostic method for LPR is 24-h multichannel intraluminal impedance-pH (MII-pH) monitoring, revealing reflux episodes irritating the upper and lower respiratory tract mucosa. The treatment of LPR should be initiated with dietary and lifestyle measures, followed by proton pump inhibitor (PPI) therapy and other measures. Despite progress, more research is needed for accurate diagnosis and targeted therapies. Key areas for exploration include biomarkers for diagnosis, the impact of non-acid reflux on symptom development, and the efficacy of new drugs. Further studies with a focused population, excluding other causes like asthma, and using new diagnostic criteria for LPR are essential. It’s crucial to consider LPR as a potential cause of unexplained chronic cough and to approach diagnosis and treatment with a multidisciplinary perspective.

## 1 Introduction

Chronic cough is defined as a non-productive cough persisting for more than eight weeks (1). That affects approximately 10% of the population and is a common reason for outpatient medical consultation (2). Chronic cough can significantly impair a patient’s quality of life, affecting physical, social, and psychological wellbeing (disruptions in sleep, reduced work productivity, social isolation, anxiety, depression and frustration etc.) (3). Cough, without expectoration, most often occurs in bronchial asthma, gastroesophageal reflux, LPR, chronic rhinosinusitis, postnasal drip syndrome, or its aetiology is unknown. Gastroesophageal reflux (GER) is considered the primary cause of chronic cough in non-smokers and non-asthmatic individuals (4). However, the understanding mechanism of LPR’s impact has led to changes in diagnostic and treatment approaches. LPR differs from typical GERD by involving gastric content reaching the sensitive larynx and pharynx (5). The exact link between LPR and chronic cough is not fully understood, but factors like direct irritation, heightened cough sensitivity, and neural reflexes likely play a role (6).

## 2 Materials and methods

Using Boolean logic, an electronic search of studies from medical and biomedical databases for the period 2010–2023 was performed. The following databases were used: PubMed, Cochrane Library, Web of Science, Scopus and Semantic Scholar. Electronic search in English language was done using following strategy: “(laryngopharyngeal reflux) OR (extraesophageal reflux) AND (chronic cough) OR (non-productive cough).” Articles without abstract were excluded. Analysis and division of the obtained publications was carried out according to their main focus and study design, i.e., whether they dealt with epidemiological-logical, pathophysiological, clinical, diagnostic or treatment issues. The main criteria according to which the publications were evaluated and divided were the population studied (adults and paediatric population), the diagnostic method of reflux (i.e., MII-pH impedance, questionnaires, clinical findings), the aim of the study and finally the results and their use in clinical practice. During the search of publications by keywords, it was found that most publications deal with gastroesophageal reflux, not laryngopharyngeal reflux or extraoesophageal reflux. Therefore, in concordance with aim of this study only articles where laryngopharyngeal reflux or extraesophageal reflux was confirmed by using dual channel intraluminal impedance (MII)/pH-metry or diagnosing with LPR questionnaires or clinical findings consistent with diagnosis of LPR were included in further analysis.

## 3 Results

**Figure 1:**
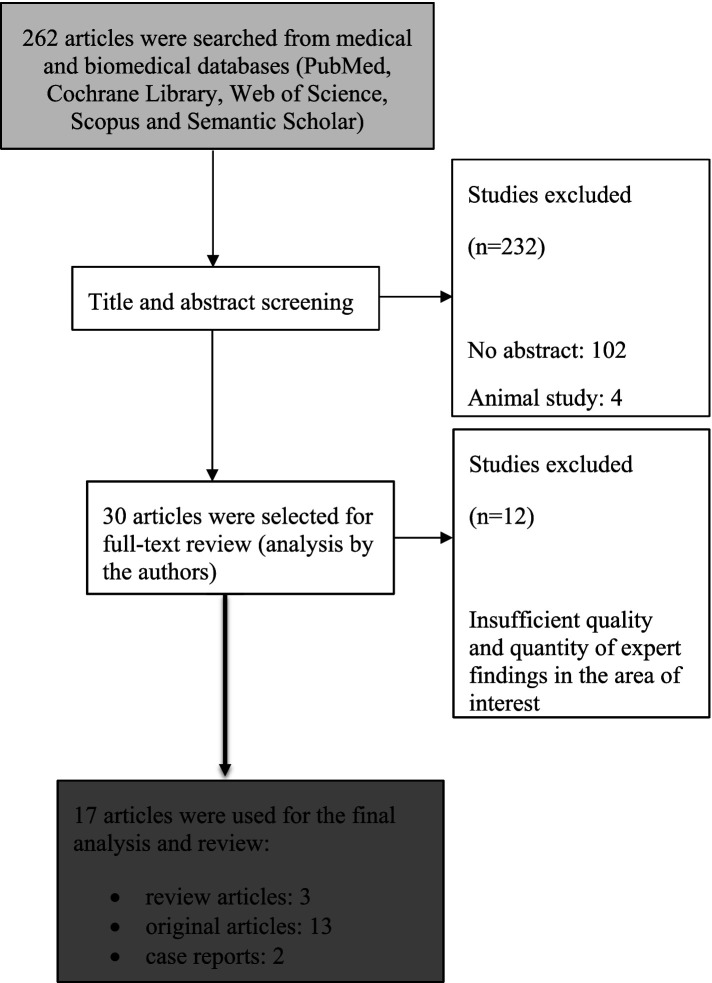
Search and selection strategy.

Our search strategy (Figure 1) yielded 262 articles in total that were subsequently screened by title and abstract. In the first step of article selection, 102 were excluded for missing abstracts, another 28 articles were excluded for describing the relationship between GER and chronic cough without detection of proximal reflux episodes (above the upper oesophageal sphincter). Another 4 articles were excluded as they were animal studies. Lastly, 98 articles were excluded as they were irrelevant to the scope of this article. Finally, 30 publications were selected for full-text review. After a thorough analysis of the articles by the authors, another 12 articles were excluded due to insufficient quality and quantity of expert findings in the area of interest. In the end, 18 articles were used for the final analysis and review (Table 1).

**Table 1:** A summary of all original articles on LPR a chronic cough that were published in the past 12 years (2010–2023).

Study	Design	LPR diagnostic modality	Subject	Result/conclusion
Kahrilas and Howden (6)	Expert panel report, systematic review	–	Effect the reflux therapy in chronic cough symptoms	Diet modification and weight loss had better cough outcomes
Wu et al. (7)	Systematic review	24-h pH impedance	Explain the mechanism of cough development in reflux.	GER is associated with increased sensitivity of the cough reflex and the development of neurogenic airway inflammation
Jiang et al. (8)	Retrospective study	24-h pH detection system	Study the value of airway pH monitoring in determining the association between chronic cough and laryngopharyngeal reflux (LPR) in children	LPR is highly associated with the development of chronic cough
Xu et al. (9)	Prospective study	24-h pH impedance	Explore association between nonacid reflux and chronic cough	Nonacid reflux is often presented with a chronic dry cough
Kikuchi et al. (10)	Case report	24-h pH monitoring	Case presentation	Chronic cough was caused by LPR in combination with silent aspiration and epiglottic inversion dysfunction
Decalmer et al. (11)	Prospective study	24-h pH impedance	Investigate the relationship between microaspiration, the degree and type of gastroesophageal reflux, and the frequency of coughing	Proximal gastroesophageal reflux and microaspiration into the airways have limited roles in provoking chronic cough
Smith and Houghton (12)	Review	24-h pH monitoring	Investigate the mechanisms thought to link LPR and cough	There is higher acid exposure in the proximal and distal esophagus of chronic cough patients compared with those without cough
Li et al. (13)	Prospective study	24-h pH impedance	Determine the mechanism of reflux-induced cough by synchronous monitoring of reflux episodes, esophageal motility, and cough	Proximal acidic reflux and distal reflux-reflex are jointly associated with reflux-induced cough
Vardar et al. (14)	Retrospective study	high-resolution manometry (HRM) and 24-h pH monitoring	Evaluation of pharyngeal and esophageal motility in chronic cough patients	HRM revealed changes to UES and esophageal motility in patients with chronic cough that are associated with impaired bolus clearance
Yu et al. (15)	Prospective study	24-h impedance and pH monitoring, Reflux symptom index (RSI)	Investigate the reflux characteristics in patients with gastroesophageal reflux-related chronic cough complicated by laryngopharyngeal reflux	In patients with higher RSI scores was presented more proximal reflux, non-acid reflux, and gas reflux, and get better efficacy with neuromodulators
Lee et al. (16)	Prospective study	24-h pH impedance	Evaluate the diagnostic usefulness of multichannel intraluminal impedance combined with pH monitoring	Reflux episodes at the distal and proximal esophagus were noted to be important factors associated with chronic cough
Ummarino et al. (17)	Diagnostic study	oropharyngeal pH-metry and 24-h pH impedance	Comparison of pH impedance monitoring and oropharyngeal (OP) pH monitoring results in patients with chronic cough	OP pH metry detected less reflux episodes than pH impedance, time correlation between cough and reflux could not be demonstrated with OP pH metry
Spyridoulias et al. (18)	Prospective study	Reflux symptom index (RSI), Reflux finding score (RFS), salivary pepsin, 24-h pH impedance	Comparison of symptoms, laryngeal signs and salivary pepsin as potential diagnostic methods for identifying LPR in patients with chronic cough	Salivary pepsin may be used as a screening adjunct to supplement the RFS in patients with extra-oesophageal symptoms
Heather et al. (19)	Retrospective study	24-h pH impedance	Investigate the relationship between chronic cough and LPR	LPR may be a prevalent contributing or etiologic factor for chronic cough. The improvement after initiating reflux treatment is 60% at 3 months
Dowgiałło-Gornowicz et al. (20)	Prospective study	24-h pH monitoring, esophagogastroduodenoscopy and otorhinolaryngological examination	Describe the results of laparoscopic Nissen fundoplication for the relief of GERD-related cough	Complete resolution of chronic cough after surgery
Chen et al. (21)	Retrospective study	24-h pH impedance	Investigate the predictive effect of positive reflux-cough correlation on the resolution of reflux-related chronic cough after anti-reflux surgery.	Laparoscopic fundoplication is effective for the management of reflux-related chronic cough
Park et al. (22)	Clinical randomized trail	Diagnostic-terapeutic test (proton pump inhibitors)	Investigation of the effectiveness and appropriate dose of PPI therapy in chronic cough	The study support the empirical use of the standard dose of PPI for 8 weeks in patients suffering from unexplained chronic cough regardless of whether reflux is present
Kopka et al. (23)	Case report	Oropharyngeal pH-metry	Case presentation	Chronic cough was caused by LPR

### 3.1 Current definitions of chronic cough and laryngopharyngeal reflux

#### 3.1.1 Chronic cough

Chronic cough, defined as a cough persisting for more than eight weeks, is a common and bothersome symptom that can significantly impact a patient’s quality of life. A persistent chronic cough can be the first sign of a more serious disease. The incidence of chronic cough is estimated to range from 9 to 33% in Europe (7). The most common causes of chronic cough in adults include angiotensin-converting enzyme inhibitor use, bronchial asthma, environmental triggers, GER, LPR and smoking. Although chronic cough is often cited as a symptom of GERD, the likelihood of direct etiology is significantly lower compared to typical symptoms such as heartburn, regurgitation, or chest pain (24). In the paediatric population, the main cause is asthma, less often GERD or LPR. In the clinical study by Koufman et al. (5), 160 patients were divided according to diagnosis: chronic cough, laryngeal stenosis, globus pharyngeus, and others. Thirty patients had chronic cough. All underwent a 24-h pH monitoring. Reflux into the throat area was detected in 52% of patients with chronic cough (25). The association between chronic cough and LPR was established, with studies suggesting that up to 10% of chronic cough cases are attributable to LPR (5). In 2021, Jiang et al. designed the study, where the association between chronic cough and LPR was analyzed. The incidence rate of LPR was 36.1% (8). The development of chronic cough based on the passage of refluxate above the upper esophageal sphincter and its impact on the respiratory system mucosa is explained from a pathophysiological perspective by the hypersensitivity of intraepithelial receptors. The most important receptors involved in the cough reflex are Aδ-fiber receptors and C-fiber receptors of the bronchial system. This refers to the sensory branch of the vagus nerve, which extends its innervation to broad regions. Generally, receptors located in the larynx region are primarily responsive to mechanical stimuli, whereas those in the distal airway are predominantly sensitive to chemical stimuli (26). Initial evaluation of the patient with a chronic cough should include a detailed history, a focused physical examination, and chest X-ray. The examination of the presence of LPR belongs mainly to an otorhinolaryngologist, who can examine mucosal changes in the pharynx and larynx and perform pH studies using protocols for detecting of extraesophageal reflux episodes. Other special diagnostic methods are indicated in a multidisciplinary manner. Treatment of chronic cough is primarily causal. Often multiple factors are involved in the development of a chronic cough, therefore, a multidisciplinary approach is necessary.

#### 3.1.2 Laryngopharyngeal reflux

LPR is defined as back flow of gastric or duodenal contents above the level of the upper esophageal sphincter. Etiopathogenesis of symptoms and diseases caused by LPR partially coincides with the pathogenesis of GERD, but it differs in many ways too. Mainly, larynx and pharynx are more sensitive to reflux components (5). As a result of the weaker defenses of the mucosa of the upper respiratory tract, chemical inflammation of the mucous membranes and tissue damage occurs with a much smaller number of reflux episodes and also as a result of less acidic (pH 4–7) reflux episodes. In addition, a significant negative effect of gastric enzymes, especially pepsin, on the tissues of the oral cavity, pharynx and respiratory tract has been demonstrated (27). The symptoms of LPR are non-specific, of varying intensity and depend on the location of the pathological action of the refluxate. LPR is usually presented with a variety of symptoms such as hoarseness, voice fatigue, burning sensation in the throat, persistent cough, sore throat, dysphagia, the sensation of a lump in the throat, and chronic throat clearing (28). The most significant difference is that the majority of patients with LPR do not have esophagitis or heartburn (29). The diagnosis of LPR is based on clinical symptoms, physical examination, therapeutic tests using diet, lifestyle modifications and proton pump inhibitors, 24-h multichannel intraluminal impedance-pH (MII-pH) monitoring and detection of pepsin (30). Treatment strategies of LPR include dietary and regimen measures, physiotherapy and diaphragm rehabilitation, proton pump inhibitors, prokinetics and alginate/malgrinate. In refractory cases, surgical intervention, such as fundoplication, may be considered (31).

### 3.2 Pathophysiology of chronic cough related to LPR

Multiple mechanisms how LPR causes chronic cough has been discusses. Research findings repeatedly showed that there are three pathophysiological mechanisms of chronic cough in association with the reflux of stomach contents into the esophagus, including direct irritation of the larynx and pharynx by gastric contents (airway reflux and microaspiration theory), increased cough sensitivity, and neural-mediated reflexes (reflex theory) and esophageal dysmotility (9). All mechanism can combine with each other.

#### 3.2.1 Proximal reflux and micro-aspiration

Proximal reflux and micro-aspiration consider that the cough-inducing stimulus may occur by direct irritation of the airways by micro-aspirations from the proximal esophagus. The presence of LPR and micro-aspiration of gastric contents into the larynx in a patient with chronic cough was described by Kikuchi et al. (10). LPR was diagnosed using 24-h pH measurement and inducted sputum culture test showed normal phagocytized bacterial flora, which suggested chronic aspiration (10). On the other hand, some studies have found no differences in proximal reflux episodes, presence of pepsin, or bile acids between chronic cough patients and control groups. Moreover, cough seems to serve a protective function for individuals with persistent cough by reducing pepsin levels in the respiratory system (11).

#### 3.2.2 Protective pharyngeal and laryngeal reflexes

Acid reflux can cause coughing through its direct impact on the upper portion of the esophagus and the laryngopharyngeal regions, or indirectly by stimulating the vagus nerve due to the influence of gastric contents on the lower esophageal part (12). In up to 75% of reflux-induced cough cases, typical acid reflux symptoms like heartburn might be missing (32). Acid plays a crucial role in the development of cough and directly activates vagal bronchopulmonary sensory nerves responsible for regulating the cough reflex. Authors Kolarik et al. described that in protecting against aspiration and inhaled irritants, Aδ-fiber nociceptors in the large airways are most effectively stimulated by quick acidification (33). In contrast, the acid-sensitive characteristics of C-fiber nociceptors enable continuous pH monitoring, which is likely essential in inflammation. Sensory input from the esophagus can sensitize cough pathways, but the receptors responsible for the acid sensitivity of vagal sensory nerves are not yet fully understood. The role of transient receptor potential cation channel subfamily V member 1 (TRPV1) has been confirmed, but the roles of acid-sensing ion channels (ASIC) and other receptors require further investigation (34).

Chronic cough can be understood as somewhat of an exaggerated defensive reaction of the pharyngolaryngeal mucosa. Of course, acute coughing itself is one of the main protective reflexes of the respiratory tract, aiming to prevent and defend the mucosa against toxic noxious agents. However, when irritation persists excessively, dysregulation occurs. Besides coughing, there are other protective reflexes as well. These reflexes consist of laryngeal closure, laryngospasm, apnea, expiration reflex, and swallowing reflex (35).

Overall, the development of chronic cough is the result of an imbalance between pathological and protective processes. Cough is a major protective laryngeal reflex, while the pharyngeal reflex involves glottal closure and contraction of the upper esophageal sphincter. Chronic cough therefore arises in case of abnormal activation of laryngopharyngeal reflexes on the basis of pathological changes in the mucous membrane caused by LPR.

#### 3.2.3 Esophageal dysmotility

In 2019, Li et al. investigated the effect of changes in peristalsis and oesophageal pressure on the development of chronic cough (13). They found that more patients had low pan-esophageal pressure in primary peristalsis and synchronous contraction in secondary peristalsis during prolonged exposure to acid in the patients with chronic cough and LPR. A study by Vardar et al. revealed changes in the motility of the proximal esophagus in patients with chronic cough using high-resolution manometry. These changes were most pronounced in patients with LPR symptoms (14).

### 3.3 Diagnostic

Diagnosing LPR-induced chronic cough can be challenging, as symptoms often overlap with other causes of chronic cough, such as asthma, postnasal drip, and gastroesophageal reflux. The lack of a gold standard diagnostic test further complicates the identification of LPR as the underlying cause. Currently, common diagnostic methods include questionnaires, laryngoscopy to identify laryngeal signs suggestive of LPR, such as erythema, edema, and postcricoid hyperplasia, and 24-h pH monitoring of the pharynx and esophagus. However, all methods have limitations in terms of sensitivity and specificity.

#### 3.3.1 Symptoms

It is important to stress out that many patients do not have the classic symptoms seen in GERD (e.g., heartburn, regurgitation). Symptoms of LPR can be assessed using the questionnaire Reflux symptom index (RSI) or Reflux Symptom Score (RSS) (36). Both are self-administered questionnaires that assesses the frequency and severity reflux-related symptoms, including chronic cough, throat clearing, and hoarseness (37). In a recent study by Yiming Yu et al. explored that RSI scores in patients with LPR related chronic cough were significantly higher than those with atopic cough or asthma induced cough (15).

#### 3.3.2 Clinical findings

A crucial examination in diagnostics of LPR is laryngoscopy, which shows changes in the larynx. Hypertrophy or redness of the posterior commissure, arytenoid processes, diffuse swelling of the larynx and pseudosulcus are often present. In cases where symptoms are severe and prolonged, laryngoscopy becomes necessary to rule out other laryngeal conditions, including the possibility of neoplasms (38). The Reflux Finding Score (RFS) and Reflux Sign Assessment (RSA) (39) are scales used by clinicians to evaluate the mucosal findings of the larynx (RFS) or larynx and other upper respiratory tract areas (RSA).

#### 3.3.3 24-h multichannel intraluminal impedance-pH (MII-pH) monitoring

MII-pH is currently considered to be gold standard for diagnosis of LPR. The pH impedance monitoring method has the ability to identify reflux episodes that are weakly acidic or non-acidic, in addition to acid reflux (16). Substantial difference with diagnosis of GERD is placing of sensors during pH-impedance studies. Most proximal sensor must be above level of upper oesophageal sphincter. Also debate regarding threshold of pathological number of extraoesophageal reflux episodes was terminated by IFOS Dubai consensus recently (40). LPR expert panel agreed that more than one extraoesophageal reflux episode is considered to by pathological and therefore threshold for LPR diagnosis.

#### 3.3.4 ResTech dx-pH measurement system

Additional method used to evaluate LPR includes the Restech system. It is a system for pH measuring in the oropharynx, i.e., above the upper oesophageal sphincter. The probe is introduced through the nose to the level of the soft palate. Unlike conventional pH electrodes, it can operate effectively in an air-filled environment. Discussed disadvantage of this method is missing oesophageal sensor and therefore ambiguous interpretation of pH drops. The study conducted by Ummarino et al. comparing the Restech system with simultaneous esophageal MII/pH monitoring revealed that most drops in pharyngeal pH are not correlated with oesophageal reflux events (17). According to the authors, it can be explained by the fact that swallow artifacts are likely to be a particular problem in patients with chronic cough who frequently sip fluids to suppress their coughing.

#### 3.3.5 Detection of pepsin

Pepsin in secretions, as a highly suggestive marker of LPR, can be detected by many methods. Detecting of pepsin using Peptest is widely used in clinical practice. Recent studies show that a positive Peptest indicates pathological LPR, but a negative Peptest does not rule out the disease (41). Spyridoulias et al. demonstrated that the identification of salivary pepsin, even at minimal levels, exhibited a baseline sensitivity of 78% and a specificity of 53% in predicting patients presenting with laryngeal signs and symptoms associated with LPR. It was demonstrated in the study that patients with severe LPR symptoms exhibit a higher concentration of salivary pepsin, and a direct association was observed between the presence and concentration of salivary pepsin and the severity of upper airway symptoms (18).

#### 3.3.6 Esophageal high-resolution manometry (HRM)

Esophageal manometry evaluates the movement patterns of the esophagus by measuring the strength of muscular contractions within it and its sphincters over time. In contrast to conventional manometry, where pressure is measured at different locations along a catheter, HRM employs a catheter equipped with numerous closely positioned pressure sensors. This setup enables the creation of a comprehensive, three-dimensional representation of pressure dynamics within the esophagus. This examination allows for the assessment of changes in the contractility of the upper esophageal sphincter, thereby helping to further elucidate the presence of laryngopharyngeal reflux. Sikavi et al. (42) discovered that reduced contractility in the upper esophagus was linked to higher levels of pharyngeal reflux in individuals experiencing symptoms of (LPR). This connection was more pronounced in patients with maintained contractile function in the lower esophagus. These results indicate that compromised contractile function in the upper esophagus may contribute to the development of LPR, as it reduces the clearance of swallowed substances or reflux, resulting in greater exposure of the laryngopharynx to refluxed material.

#### 3.3.7 Diagnostic test (empiric therapy of PPI’s)

The results of Park et al. study support the empirical use of the standard dose of PPI for 8 weeks in patients suffering from unexplained chronic cough regardless of whether reflux is present (22). The use of empiric therapy of PPI’s to both diagnose and treat reflux-associated chronic cough has also been studied by Poe et al. The study revealed that 79% of patients with reflux-related cough were accurately diagnosed, and their symptoms were alleviated following an empirical trial of PPI therapy (43).

PPIs are the first choice in GERD therapy, but their efficacy is somewhat limited in the case of LPR. There are several theories as to why this occurs. LPR frequently includes reflux events that are either non-acidic or weakly acidic. Since PPIs mainly aim at decreasing stomach acid production, they might not be as efficient in alleviating symptoms stemming from non-acidic reflux (44). The primary function of PPIs is to reduce the production of hydrochloric acid in the stomach, thereby decreasing its quantity. However, PPIs do not affect its effects in locations such as the larynx or pharynx, which leads to the development of symptoms in these areas. PPIs have no effect on the intracellular activity of pepsin, which can independently irritate the mucous membranes of the upper respiratory tract (45). Given the limitations of PPIs in treating LPR, other treatment modalities may be more appropriate. Since dietary and lifestyle measures are indispensable parts of LPR therapy, it might be more advantageous to conduct a diagnostic-therapeutic test using a diet instead of PPIs. This is supported by the research conducted by Lechien et al. (46), who suggest that adopting a diet low in fat, quick-release sugars, and high in protein, alkaline foods, and plant-based foods could serve as a cost-effective alternative therapy for individuals with LPR.

### 3.4 Treatment

Treatment of LPR-related chronic cough typically involves lifestyle modifications, such as dietary changes, weight loss, and avoiding triggers like smoking and alcohol. Additionally, medical therapy with proton pump inhibitors (PPIs) or alginate/magaldrate (ideally on the basis of results of pH-impedance) can be effective in reducing acid reflux and improving symptoms (47). Surgery is reserved exclusively for severe cases where conventional pharmacotherapy fails to achieve the desired results. It is important to note that LPR is just one potential cause of chronic cough, and a thorough evaluation by a healthcare professional is necessary to determine the other possible causes.

#### 3.4.1 Lifestyle modifications

Lifestyle modifications and diet are principal treatment modalities. It’s particularly important to thoroughly and carefully explain to the patient the specific measures and provide them with precise instructions. Recommendations include weight reduction, smoking cessation, elevating the upper body while sleeping, stress reduction, avoiding late-night eating, and consuming meals in smaller portions. As part of the anti-reflux diet, it’s important to educate the patient that even healthy foods can be unsuitable for treating reflux, for example tomatoes, onions, garlic, nuts. Also, it is essential to limit the intake of alcoholic beverages, caffeine, chocolate, fatty and spicy foods, juices and carbonated drinks. An anti-reflux diet is the primary treatment approach for the majority of patients (48). In study conducted by Smith et al. was demonstrated that there is a strong correlation between high calorie and fat intake and the severity of cough symptoms (49). Heather Yeakel et al. reported a subjective improvement in chronic cough in 60% of patients with LPR during dietary and lifestyle therapy (19).

#### 3.4.2 Physiotherapy, diaphragm rehabilitation and speech therapy

The objective of the exercise is to enhance the functionality of the diaphragm, resulting in a decrease in the frequency of reflux episodes (50). The lower esophageal sphincter (LES), surrounded by the diaphragmatic muscle, prevents gastroesophageal reflux and indirectly, laryngopharyngeal reflux (LPR). The coordinated function of the LES and its surrounding diaphragmatic crura is crucial for proper closure. When these structures become ineffective, gastric contents can flow back along the esophagus and cause LPR (51). Experimental studies show that even after surgical removal of the LES, a pressure zone remains due to diaphragmatic crura contractions (52). Strengthening the diaphragm muscle through exercises, such as diaphragmatic muscle training with maneuvers and breathing exercises, is considered in alternative therapies for reflux disease.

Speech therapy has shown effectiveness for chronic cough. In one study of 20 patients with cough and paradoxical vocal fold movement disorder, treated with a PPI and respiratory retraining therapy, all experienced cough improvement, with 85% showing improved RSI (53).

#### 3.4.3 Proton pumps inhibitors (PPIs)

Among the available medications, they are the most commonly utilized and exert the most notable impact. However, their efficacy in addressing extraesophageal manifestations is relatively limited compared to the treatment of esophageal reflux disease. Proton pump inhibitors (PPIs) primarily aim to reduce acidity rather than significantly prevent reflux. Proton pump inhibitor (PPI) therapy, administered at a dosage of up to twice daily for 8–12 weeks, should be considered for patients who have both esophageal reflux symptoms and concomitant LPR, or for those who have confirmed pathological reflux through objective testing. However, there is currently a lack of adequate evidence to support the use of empiric PPI therapy in patients who solely experience LPR symptoms (54). Several uncontrolled studies have indicated that treating chronic cough with antacids can lead to improvement (55). However, recent randomized controlled trial (56) has demonstrated no discernible distinctions between proton pump inhibitors (PPIs) and a placebo in terms of their effectiveness.

#### 3.4.4 Alginate, magaldrate

Are recommended for weakly acid and alkaline LPR. After ingestion, alginate/magaldrate quickly reacts with stomach acid and forms a protective gel layer.

#### 3.4.5 Prokinetics

Prokinetics support the peristalsis by activating receptors in the gut, known as dopamine or serotonin receptors and thereby reduce the risk of reflux. Through this activation, prokinetics encourage synchronized muscle contractions, enhancing the efficient transit of food and digestive materials through the digestive tract. The effect in the treatment of EER is questionable. It is known that prokinetics increase esophageal peristaltic contractions, thereby facilitating the movement of esophageal contents into lower levels of the digestive system (57). Some studies suggest that the combination of PPIs and prokinetics yields superior outcomes and efficacy compared to the use of PPIs alone (58). The debate surrounding the effectiveness of prokinetics in LPR underscores the dearth of evidence regarding the occurrence of esophageal dysmotility disorder in this condition (59).

#### 3.4.6 Surgical therapy

The most frequently performed surgical procedure for treating reflux disease is laparoscopic fundoplication. It involves wrapping a section of the stomach around the esophagus, forming a cuff that secures the stomach in its proper position under the diaphragm. This technique effectively prevents the backflow of stomach contents into the esophagus and is widely utilized in surgical interventions for reflux disease. Anti-reflux surgery may lead to improvements in syndromes of chronic cough (20, 21), laryngopharyngeal reflux, and asthma, but only in highly selected patients (60). It is important to note that the available data on this subject are generally of low quality. Furthermore, there are currently no randomized controlled trials comparing anti-reflux surgery with medical therapy specifically for the treatment of cough or laryngopharyngeal reflux. Considering the smaller impact of surgery on treating of LPR compared to gastroesophageal reflux disease, it is essential to exercise caution when determining the indication for surgical intervention (61).

### 3.5 Impact on quality of life

Chronic cough due to LPR can significantly impair a patient’s quality of life, affecting physical, social, and psychological wellbeing (19). Patients may experience disruptions in sleep, reduced work productivity, and social isolation due to the persistent nature of their cough. Furthermore, the impact on mental health should not be underestimated, as the chronic nature of the condition can lead to anxiety, depression, and frustration. With the development of the biological-psychological-social medical model, several questionnaires were created that focused on the evaluation of the quality of life in a relationship with chronic cough and LPR (e.g., Cough-specific Quality-of-life Questionnaire (CQLQ) and the Laryngopharyngeal Reflux Health Related Quality of Life Questionnaire (LPR-HRQL)). Successful treatment can not only relieve cough symptoms but also improve the quality of life (QOL) of these patients (62).

### 3.6 Patient education and multidisciplinary approach

A multidisciplinary approach involving primary care physicians, gastroenterologists, otolaryngologists, pulmonologists, and speech-language therapists can help ensure comprehensive care and optimal outcomes for patients with LPR-induced chronic cough. Collaboration among healthcare professionals is essential for accurate diagnosis, individualized treatment plans, and ongoing monitoring of patients’ progress. Depending on the physician’s specialty and experience, LPR may be over- or under-diagnosed (63). Educating patients about LPR and chronic cough is crucial for successful management. Patients should understand the importance of adhering to lifestyle modifications and prescribed medications to alleviate symptoms and prevent complications (54). Additionally, they should be informed about the potential side effects of medications and when to seek medical attention for any concerns.

## 4 Conclusion

Chronic cough is one of possible manifestations of LPR. Studies indicate that approximately 20% of patients with chronic cough have diagnosed LPR. The main diagnostic method for examining and confirming LPR is considered to be a 24-h multichannel intraluminal impedance-pH (MII-pH) monitoring. This method clearly demonstrates reflux episodes that reach above the level of the upper esophageal sphincter, leading to irritation of the mucosa of the upper and lower respiratory tracts by gastric refluxate. Treatment of LPR in patients with chronic cough should start with dietary and lifestyle measures, followed by initiating PPI therapy and others. Despite advancements in our understanding of LPR and its association with chronic cough, there remains a need for more research to improve diagnostic accuracy and develop targeted therapies. Potential areas of exploration include the role of biomarkers in LPR diagnosis, the impact of non-acid reflux components (e.g., bile acids and pepsin) on symptom development, and the efficacy of novel pharmacologic agents in LPR management (64). Further studies are needed with a precisely defined monitored population (patients with chronic cough excluding bronchial asthma and other possible causes) in which a complete diagnosis of LPR will be performed with a uniform methodology. This is also supported by the fact that LPR is a relatively young term and most studies so far deal with gastroesophageal reflux. It is important to think of LPR as a cause of unexplained chronic cough. A correct diagnostic and therapeutic process can only be achieved with a multidisciplinary approach.

## Author contributions

VH: Writing – original draft, Methodology. TB: Writing – original draft, Methodology. PG: Writing – review & editing, Data curation. LS: Writing – review & editing, Methodology. KZ: Writing – review & editing, Supervision, Conceptualization. PK: Writing – review & editing, Supervision.

## References

[1] Morice A Dicpinigaitis P McGarvey L Birring SS. Chronic cough: new insights and future prospects. Eur Respir Rev. (2021) 30:210127. doi: 10.1183/16000617.0127-2021, PMID: 34853095 PMC9488126

[2] Krüger K Holzinger F Trauth J Koch M Heintze C Gehrke-Beck S. Chronic cough. Dtsch Arztebl Int. (2022) 119:59–65. doi: 10.3238/arztebl.m2021.0396, PMID: 34918623 PMC9059861

[3] Irwin RS Madison JM. The diagnosis and treatment of cough. N Engl J Med. (2000) 343:1715–21. doi: 10.1056/NEJM200012073432308 11106722

[4] Sylvester DC Karkos PD Vaughan C Johnston J Dwivedi RC Atkinson H. Chronic cough, reflux, postnasal drip syndrome, and the otolaryngologist. Int J Otolaryngol. (2012) 2012:1–5. doi: 10.1155/2012/564852, PMID: 22577385 PMC3332192

[5] Koufman JA Aviv JE. Laryngopharyngeal reflux: update on diagnosis and treatment. Curr Opin Otolaryngol Head Neck Surg. (2015) 23:210–4.

[6] Kahrilas PJ Howden CW. Chronic cough and laryngopharyngeal reflux. Gastroenterol Hepatol. (2014) 10:663–5. doi: 10.1378/chest.12-1788

[7] Wu J Ma Y Chen Y. GERD – related chronic cough: possible mechanism, diagnosis and treatment. Front Physiol. (2020) 13:1005404. doi: 10.3389/fphys.2022.1005404, PMID: 36338479 PMC9630749

[8] Jiang YL Li D Li TT Wu BR Yin BR Li AQ. Value of airway pH monitoring in determining the association between chronic cough and laryngopharyngeal reflux in children. Zhongguo Dang Dai Er Ke Za Zhi. (2021) 23:713–7. doi: 10.7499/j.issn.1008-8830.2102022 PMID: 34266529 PMC8292663

[9] Xu X Yu L Chen Q Lv H Qiu Z. Diagnosis and treatment of patients with nonacid gastroesophageal reflux-induced chronic cough. J Res Med Sci. (2015) 20:885–92. doi: 10.4103/1735-1995.170625, PMID: 26759577 PMC4696375

[10] Kikuchi A Kawamoto R Mizumoto J Akase T Ninomiya D Kumagi T. A case of laryngopharyngeal reflux-associated chronic cough: misinterpretation of treatment efficacy causes diagnostic delay. J Gen Fam Med. (2020) 21:258–60. doi: 10.1002/jgf2.348, PMID: 33304721 PMC7689238

[11] Decalmer S Stovold R Houghton LA Pearson J Ward C Kelsall A. Chronic cough: relationship between microaspiration, gastroesophageal reflux, and cough frequency. Chest. (2012) 142:958–64. doi: 10.1378/chest.12-0044 22797535

[12] Smith JA Houghton LA. The oesophagus and cough: laryngo-pharyngeal reflux, microaspiration and vagal reflexes. Cough. (2013) 9:12. doi: 10.1186/1745-9974-9-12, PMID: 23590893 PMC3640905

[13] Li X Lin S Wang Z Zhang H Sun X Li J. Gastroesophageal reflux disease and chronic cough: a possible mechanism elucidated by ambulatory pH-impedance-pressure monitoring. Neurogastroenterol Motil. (2019) 31:e13707. doi: 10.1111/nmo.13707, PMID: 31482661 PMC6899806

[14] Vardar R Sweis R Anggiansah A Wong T Fox MR. Upper esophageal sphincter and esophageal motility in patients with chronic cough and reflux: assessment by high-resolution manometry. Dis Esophagus. (2013) 26:219–25. doi: 10.1111/j.1442-2050.2012.01354.x, PMID: 22591118

[15] Yu Y Wen S Wang S Shi C Ding H Qiu Z. Reflux characteristics in patients with gastroesophageal reflux-related chronic cough complicated by laryngopharyngeal reflux. Ann Transl Med. (2019) 7:529. doi: 10.21037/atm.2019.09.162, PMID: 31807511 PMC6861741

[16] Lee JH Park SY Cho SB Lee WS Park CH Koh YI. Reflux episode reaching the proximal esophagus are associated with chronic cough. Gut Liver. (2012) 6:197–202. doi: 10.5009/gnl.2012.6.2.197, PMID: 22570748 PMC3343157

[17] Ummarino D Vandermeulen L Roosens B Urbain D Hauser B Vandenplas Y. Gastroesophageal reflux evaluation in patients affected by chronic cough: Restech versus multichannel intraluminal impedance/pH metry. Laryngoscope. (2013) 123:980–4. doi: 10.1002/lary.23738 23023943

[18] Spyridoulias A Lillie S Vyas A Fowler SJ. Detecting laryngopharyngeal reflux in patients with upper airways symptoms: symptoms, signs or salivary pepsin? Respir Med. (2015) 109:963–9. doi: 10.1016/j.rmed.2015.05.019 26044812

[19] Heather Y Bailey B Swetha V Ghiath A Robert TS. The relationship between chronic cough and laryngopharyngeal reflux. J Voice. (2023) 37:245–50. doi: 10.1016/j.jvoice.2020.11.011, PMID: 33262000

[20] Dowgiałło-Gornowicz N Masiewicz A Kacperczyk J Lech P Saluk S Osowiecka K. Long-term outcomes of chronic cough reduction after laparoscopic Nissen fundoplication-a single-center study. Medicina (Kaunas). (2021) 58:47. doi: 10.3390/medicina58010047, PMID: 35056354 PMC8779940

[21] Chen D Wang Z Hu Z Liang Y Xiao F Wu J. Typical symptoms and not positive reflux-cough correlation predict cure of gastroesophageal reflux disease related chronic cough after laparoscopic fundoplication: a retrospective study. BMC Gastroenterol. (2019) 19:108. doi: 10.1186/s12876-019-1027-8, PMID: 31242859 PMC6595575

[22] Park HJ Park YM Kim JH Lee HS Kim HJ Ahn CM. Effectiveness of proton pump inhibitor in unexplained chronic cough. PLoS One. (2017) 12:e0185397. doi: 10.1371/journal.pone.0185397, PMID: 29016626 PMC5634560

[23] Kopka M Małecka M Stelmach I. Chronic cough as a symptom of laryngopharyngeal reflux--two case reports. Pneumonol Alergol Pol. (2016) 84:29–32. doi: 10.5603/PiAP.a2015.0082, PMID: 26687670

[24] Gyawali CP Yadlapati R Fass R Katzka D Pandolfino J Savarino E. Updates to the modern diagnosis of GERD: Lyon consensus 2.0. Gut. (2024) 73:361–71. doi: 10.1136/gutjnl-2023-330616 37734911 PMC10846564

[25] Fraser-Kirk K. Laryngopharyngeal reflux: a confounding cause of aerodigestive dysfunction. Aust Fam Physician. (2017) 46:34–9. PMID: 28189129

[26] Canning J. Afferent nerves regulating the cough reflex: mechanisms and mediators of cough in disease. Otolaryngol Clin N Am. (2010) 43:15–25. doi: 10.1016/j.otc.2009.11.012, PMID: 20172253 PMC2882535

[27] Li Y Xu G Zhou B Tang Y Liu X Wu Y. Effects of acids, pepsin, bile acids, and trypsin on laryngopharyngeal reflux diseases: physiopathology and therapeutic targets. Eur Arch Otorrinolaringol. (2022) 279:2743–52. doi: 10.1007/s00405-021-07201-w, PMID: 34860271 PMC9072476

[28] Koufman JA. The otolaryngologic manifestations of gastroesophageal reflux disease (GERD): a clinical investigation of 225 patients using ambulatory 24-hour pH monitoring and an experimental investigation of the role of acid and pepsin in the development of laryngeal injury. Laryngoscope. (1991) 101:1–78. doi: 10.1002/lary.1991.101.s53.1 1895864

[29] Toohill RJ Kuhn JC. Role of refluxed acid in pathogenesis of laryngeal disorders. Am J Med. (1997) 103:100S–6S. doi: 10.1016/S0002-9343(97)00333-1 9422633

[30] Junaid M Qadeer Ahmed S Kazi M Khan HU Sohail HM. Laryngopharyngeal reflux disease: outcome of patients after treatment in otolaryngology clinics. Cureus. (2020) 12:e12195. doi: 10.7759/cureus.12195, PMID: 33489604 PMC7816050

[31] Broeders JA Mauritz FA Ahmed Ali U Draaisma WA Ruurda JP Gooszen HG. Systematic review and meta-analysis of laparoscopic Nissen (posterior total) versus Toupet (posterior partial) fundoplication for gastro-oesophageal reflux disease. Br J Surg. (2010) 97:1318–30. doi: 10.1002/bjs.7174, PMID: 20641062

[32] Alhajjaj MS Bajaj P. Chronic cough. In: StatPearls [internet]. Treasure Island (FL): StatPearls Publishing; (2023). Available at: https://www.ncbi.nlm.nih.gov/books/NBK430791 (Accessed May 8, 2022).

[33] Kollarik M Fei R Undem BJ. Acid-sensitive vagal sensory pathways and cough. Pulm Pharmacol Ther. (2007) 20:402–11. doi: 10.1016/j.pupt.2006.11.010, PMID: 17289409 PMC2577168

[34] Canning BJ Mori N Mazzone SB. Vagal afferent nerves regulating the cough reflex. Respir Physiol Neurobiol. (2006) 152:223–42. doi: 10.1016/j.resp.2006.03.001 16740418

[35] Ludlow CL. Laryngeal reflexes: physiology, technique, and clinical use. J Clin Neurophysiol. (2015) 32:284–93. doi: 10.1097/WNP.0000000000000187, PMID: 26241237 PMC4527097

[36] Lechien JR Bobin F Muls V Thill MP Horoi M Ostermann K. Validity and reliability of the reflux symptom score. Laryngoscope. (2020) 130:98–107. doi: 10.1002/lary.28017 30983002

[37] Belafsky PC Postma GN Koufman JA. Validity and reliability of the reflux symptom index (RSI). J Voice. (2002) 16:274–7. doi: 10.1016/S0892-1997(02)00097-8 12150380

[38] Campagnolo AM Priston J Thoen RH. Laryngopharyngeal reflux: diagnosis, treatment, and latest research. Int Arch Otorhinolaryngology. (2014) 18:184–91. doi: 10.1055/s-0033-1352504, PMID: 25992088 PMC4297018

[39] Lechien JR Rodriguez Ruiz A Dequanter D Bobin F Mouawad F Muls V. Validity and reliability of the reflux sign assessment. Ann Otol Rhinol Laryngol. (2020) 129:313–25. doi: 10.1177/0003489419888947 31729247

[40] Lechien JR Vaezi MF Chan WW Allen JE Karkos PD Saussez S. The Dubai definition and diagnostic criteria of laryngopharyngeal reflux: the IFOS consensus. Laryngoscope. (2023) 134:1614–24. doi: 10.1002/lary.31134, PMID: 37929860

[41] Zeleník K Hránková V Vrtková A Staníková L Komínek P Formánek M. Diagnostic value of the Peptest TM in detecting laryngopharyngeal reflux. J Clin Med. (2021) 10:2996. doi: 10.3390/jcm10132996, PMID: 34279479 PMC8268930

[42] Sikavi DR Cai JX Leung R Carroll TL Chan WW. Impaired proximal esophageal contractility predicts pharyngeal reflux in patients with laryngopharyngeal reflux symptoms. Clin Transl Gastroenterol. (2021) 12:e00408. doi: 10.14309/ctg.0000000000000408, PMID: 34597279 PMC8487779

[43] Poe RH Kallay MC. Chronic cough and gastroesophageal reflux disease: experience with specific therapy for diagnosis and treatment. Chest. (2003) 123:679–84. doi: 10.1378/chest.123.3.679, PMID: 12628862

[44] Mermelstein J Chait Mermelstein A Chait MM. Proton pump inhibitor-refractory gastroesophageal reflux disease: challenges and solutions. Clin Exp Gastroenterol. (2018) 11:119–34. doi: 10.2147/CEG.S121056, PMID: 29606884 PMC5868737

[45] Johnston N Dettmar PW Ondrey FG Nanchal R Lee SH Bock JM. Pepsin: biomarker, mediator, and therapeutic target for reflux and aspiration. Ann N Y Acad Sci. (2018) 1434:282–9. doi: 10.1111/nyas.13729, PMID: 29774546

[46] Lechien JR Crevier-Buchman L Distinguin L Iannella G Maniaci A De Marrez LG. Is diet sufficient as laryngopharyngeal reflux treatment? A cross-over observational study. Laryngoscope. (2022) 132:1916–23. doi: 10.1002/lary.29890, PMID: 34606102

[47] Delgaudio JM Waring JP Teixeira MS. Reflux laryngitis and its sequelae: the diagnostic role of ambulatory 24-hour pH monitoring. Ann Otol Rhinol Laryngol. (2019) 128:173–8.

[48] Koufman JA. Low-acid diet for recalcitrant laryngopharyngeal reflux: therapeutic benefits and their implications. Ann Otol Rhinol Laryngol. (2011) 120:281–7. doi: 10.1177/000348941112000501, PMID: 21675582

[49] Smith JE Morjaria JB Morice AH. Dietary intervention in the treatment of patients with cough and symptoms suggestive of airways reflux as determined by Hull airways reflux questionnaire. Cough. (2013) 9:27. doi: 10.1186/1745-9974-9-27 24380385 PMC3883462

[50] Qiu K Wang J Chen B Wang H. The effect of breathing exercises on patients with GERD: a meta-analysis. Ann Palliat Med. (2020) 9:405–13. doi: 10.21037/apm.2020.02.35, PMID: 32233626

[51] Mittal R Balaban D. The esophagogastric junction. N Engl J Med. (1997) 336:924–32. doi: 10.1056/NEJM199703273361306 9070474

[52] Klein W Parkman H Dempsey D Fisher R. Sphincterlike thoracoabdominal high pressure zone after esophagogastrectomy. Gastroenterology. (1993) 105:1362–9. doi: 10.1016/0016-5085(93)90140-8, PMID: 8224640

[53] Murry T Tabaee A Owczarzak V Aviv JE. Respiratory retraining therapy and management of laryngopharyngeal reflux in the treatment of patients with cough and paradoxical vocal fold movement disorder. Ann Otol Rhinol Laryngol. (2006) 115:754–8. doi: 10.1177/000348940611501007 17076097

[54] Martinucci I de Bortoli N Savarino E Nacci A Romeo SO Bellini M. Optimal treatment of laryngopharyngeal reflux disease. Ther Adv Chronic Dis. (2013) 4:287–301. doi: 10.1177/2040622313503485, PMID: 24179671 PMC3807765

[55] Qureshi F Asad H Patel PS Ramprasad A Singh SP Suman S. Gastroesophageal reflux disease-associated chronic cough: a population-based analysis of patient presentations in the United States. Cureus. (2021) 13:e17512. doi: 10.7759/cureus.17512, PMID: 34595079 PMC8473893

[56] Hara J Stocken DD Watson GC Fouweather T McGlashan J MacKenzie K. Use of proton pump inhibitors to treat persistent throat symptoms: multicentre, double blind, randomised, placebo controlled trial. BMJ. (2021) 372:4903. doi: 10.1136/bmj.m4903, PMID: 33414239 PMC7789994

[57] Mikami H Ishimura N Fukazawa K Okada M Izumi D Shimura S. Effects of metoclopramide on esophageal motor activity and esophagogastric junction compliance in healthy volunteers. J Neurogastroenterol Motil. (2016) 22:112–7. doi: 10.5056/jnm15130, PMID: 26507875 PMC4699728

[58] Chun BJ Lee DS. The effect of itopride combined with lansoprazole in patients with laryngopharyngeal reflux disease. Eur Arch Otorrinolaringol. (2013) 270:1385–90. doi: 10.1007/s00405-012-2341-8, PMID: 23292040

[59] Benjamin T Zackria S Lopez R Richter J Thota PN. Upper esophageal sphincter abnormalities and high-resolution esophageal manometry findings in patients with laryngopharyngeal reflux. Scand J Gastroenterol. (2017) 52:816–21. doi: 10.1080/00365521.2017.1322139, PMID: 28471304

[60] Sidwa F Moore AL Alligood E Fisichella PM. Surgical treatment of extraesophageal manifestations of gastroesophageal reflux disease. World J Surg. (2017) 41:2566–71. doi: 10.1097/SLA.0000000000001907, PMID: 28508234

[61] Francis DO Goutte M Slaughter JC Garrett CG Hagaman D Holzman MD. Traditional reflux parameters and not impedance monitoring predict outcome after fundoplication in extraesophageal reflux. Laryngoscope. (2011) 121:1902–9. doi: 10.1002/lary.21897 22024842

[62] Wang Z Wang M Wen S Yu L Xu X. Types and applications of cough-related questionnaires. J Thorac Dis. (2019) 11:4379–88. doi: 10.21037/jtd.2019.09.62, PMID: 31737324 PMC6837954

[63] Lechien JR Saussez S Muls V Barillari MR Chiesa-Estomba CM Hans S. Laryngopharyngeal reflux: a state-of-the-art algorithm management for primary care physicians. J Clin Med. (2020) 9:3618. doi: 10.3390/jcm9113618, PMID: 33182684 PMC7697179

[64] Jiang A Liang M Su Z Chai L Lei W Wang Z. Immunohistochemical detection of pepsin in laryngeal mucosa for diagnosing laryngopharyngeal reflux. Laryngoscope. (2011) 121:1426–30. doi: 10.1002/lary.21809, PMID: 21647907

